# Comparison of the conceive-design-implement-operate model and lecture-based learning in teaching the “healthcare-associated infections” course

**DOI:** 10.3389/fmed.2025.1505588

**Published:** 2025-01-22

**Authors:** Yu Chen, Rui Zhang, Zhenke Zhou, Min Hong, Zheng Huang, Heling Wen, Lei Peng

**Affiliations:** ^1^Department of Cardiology, Sichuan Provincial People's Hospital, School of Medicine, University of Electronic Science and Technology of China, Chengdu, China; ^2^Department of Surgery, The Affiliated Tumor Hospital of Chengdu Medical College, Chengdu, China; ^3^Department of Infectious Disease, The Affiliated Tumor Hospital of Chengdu Medical College, Chengdu, China; ^4^Department of Fever Clinic, Sichuan Provincial People's Hospital, Sichuan Academy of Medical Science, University of Electronic Science and Technology of China, Chengdu, China; ^5^Department of Nephrology, Sichuan Provincial People's Hospital, School of Medicine, University of Electronic Science and Technology of China, Chengdu, China

**Keywords:** healthcare-associated infections, conceive-design-implement-operate model, lecture-based learning, nursing student, teaching model

## Abstract

**Background:**

Healthcare-associated infections (HAI) are infections acquired by patients during treatment in various healthcare institutions. These infections significantly increase morbidity, mortality, and healthcare costs. Enhancing HAI education for nurses can improve patient safety and medical quality.

**Aim:**

The study aimed to assess the effectiveness of the new conceive-design-implement-operate (CDIO) teaching model on nursing students’ HAI learning outcomes and compare it with the traditional LBL model, providing valuable insights for future HAI education in nursing.

**Methods:**

A total of 110 nursing students were randomly assigned to one of two groups for HAI training during the 2022–2023 academic year: a group that engaged in the CDIO model and another that received traditional lecture-based learning (LBL). The effectiveness of these pedagogical approaches was evaluated by comparing pre-and post-training test scores, and we used the Course Experience Questionnaire (CEQ) to collect students’ feedback on the course and teaching.

**Results:**

Compared to traditional LBL method, the CDIO model significantly improved the overall scores and practical application scores of nursing students in the HAI course, with these advantages still retained after 24 weeks. Additionally, preliminary results show that students in the CDIO model scored higher on CEQ categories such as good teaching, clear goals and standards, appropriate assessment, generic skills, and independence, but they also reported an increased workload.

**Conclusion:**

Our research is the first to apply the CDIO framework to nursing education in HAI courses, enhancing nursing students’ practical application skills, particularly in the sustained retention in this area. Our study indicates that the CDIO teaching model has significant advantages in enhancing course experience and teaching effectiveness.

## Introduction

Healthcare-associated infections (HAI), also known as nosocomial or hospital-acquired infections, are infections that individuals acquire while receiving treatment for other conditions within various healthcare settings, including acute care hospitals, long-term care facilities such as nursing homes, outpatient surgical centers, dialysis centers, or ambulatory care clinics (1). Common types of HAI include central line-associated bloodstream infections, catheter-associated urinary tract infections, surgical site infections, ventilator-associated pneumonia, and so on (2).

The burden of HAI in developing countries is significantly high, with a reported pooled prevalence rate of 15.5 per 100 patients in high-quality studies, exceeding those observed in Europe and the United States (3). Furthermore, the study revealed a significantly high prevalence of HAI reaching up to 34.1% in critically ill patients in a developing country’s ICU, compounding the already observed high incidence of adverse events, which necessitates increased care and prolongs hospital stays (4). These HAIs are significant contributors to patient morbidity and mortality and represent a considerable economic burden due to increased hospital stays, additional treatments, and other healthcare costs (5). Nurses have a high risk of transmitting HAIs among patients and healthcare personnel, but studies indicate that there are still gaps in their knowledge and practice of HAI prevention (6, 7). Therefore, strengthening HAI training for nurses is of great importance. The teaching of the HAI course encompasses not only the learning of theoretical knowledge but also emphasizes the training of practical skills, such as the correct steps for hand hygiene and the proper use of personal protective equipment. It requires a close integration of theory and practice to ensure timely adjustments and improvements in educational strategies through the evaluation of educational outcomes and feedback, ultimately ensuring that educational activities effectively reduce the rate of HAI.

Lecture-based Learning (LBL), a teacher-centered method focused on knowledge transmission, was first introduced in US medical schools in 1894. Students merely receive information from teachers and attempt to memorize content, rather than understanding and applying knowledge (8–10). This LBL model, focused on memorization and one-sided knowledge transmission, is inadequate for preparing nurses for the current healthcare realities. Therefore, there is a need to promote and implement a new, innovative classroom model in nursing education that is learner-centered and competency-based (11, 12).

The Conceive-Design-Implement-Operate (CDIO) approach, developed in 2000, is a student-centered engineering education framework that fosters active learning of both theoretical knowledge and practical skills (13). In recent years, the application of the CDIO model in medical and nursing education has gradually increased. Compared with the control group, the theoretical and technical scores of the residents in the CDIO model group significantly improved in the COVID-19 Prevention Training (14). One teaching study based on the CDIO concept found that the CDIO model can promote the organic integration of theoretical and practical aspects of orthopedics clinical education for nursing students, enhance their ability to apply theoretical knowledge to analyze and solve practical problems, and improve teaching effectiveness (15). A study on online cardiovascular health training for nursing students found that the CDIO model improved students’ health education skills and clinical decision-making, outperforming traditional teaching methods in both theoretical and practical tests (16). These studies suggest that the CDIO model’s emphasis on “student-led learning” and “the integration of theory and practice” may be highly aligned with the HAI course’s goals of enhancing theoretical knowledge, problem-solving abilities, and clinical practice.

This randomized controlled study aims to explore the effectiveness of the new CDIO teaching model on learning outcomes related to HAI for nursing students, and to compare its effectiveness with that of the traditional LBL model. We hypothesize that the CDIO model is more effective than the LBL model in both theoretical and practical aspects. Additionally, we used the Course Experience Questionnaire (CEQ) to collect students’ feedback on the course and teaching.

## Methods

### Study design and participants

This study was a parallel randomized controlled trial involving two distinct groups, comprising three assessments and a quantitative questionnaire. It was carried out at a teaching and research hospital during the 2022–2023 academic year. Third-year nursing students participating in surgical internships were recruited. All participants were required to have no prior experience in learning about HAI courses. These participants were randomly assigned to one of two groups. The experimental group received training through the CDIO method, while the control group was instructed using the traditional LBL approach.

### Sample size

Before commencing the study, we performed a sample size estimation based on the results of our preliminary research. Using an alpha level of 0.05 and a power of 0.9, we determined that 50 participants per group were necessary. To account for a 10% attrition rate, the sample size for each group was adjusted to 55, bringing the total number of participants to 110. Sample size calculation was conducted using PASS 15.0 (Power Analysis and Sample Size) software (UT, United States).

### Interventions

#### The CDIO approach for HAI curriculum

**Figure 1:**
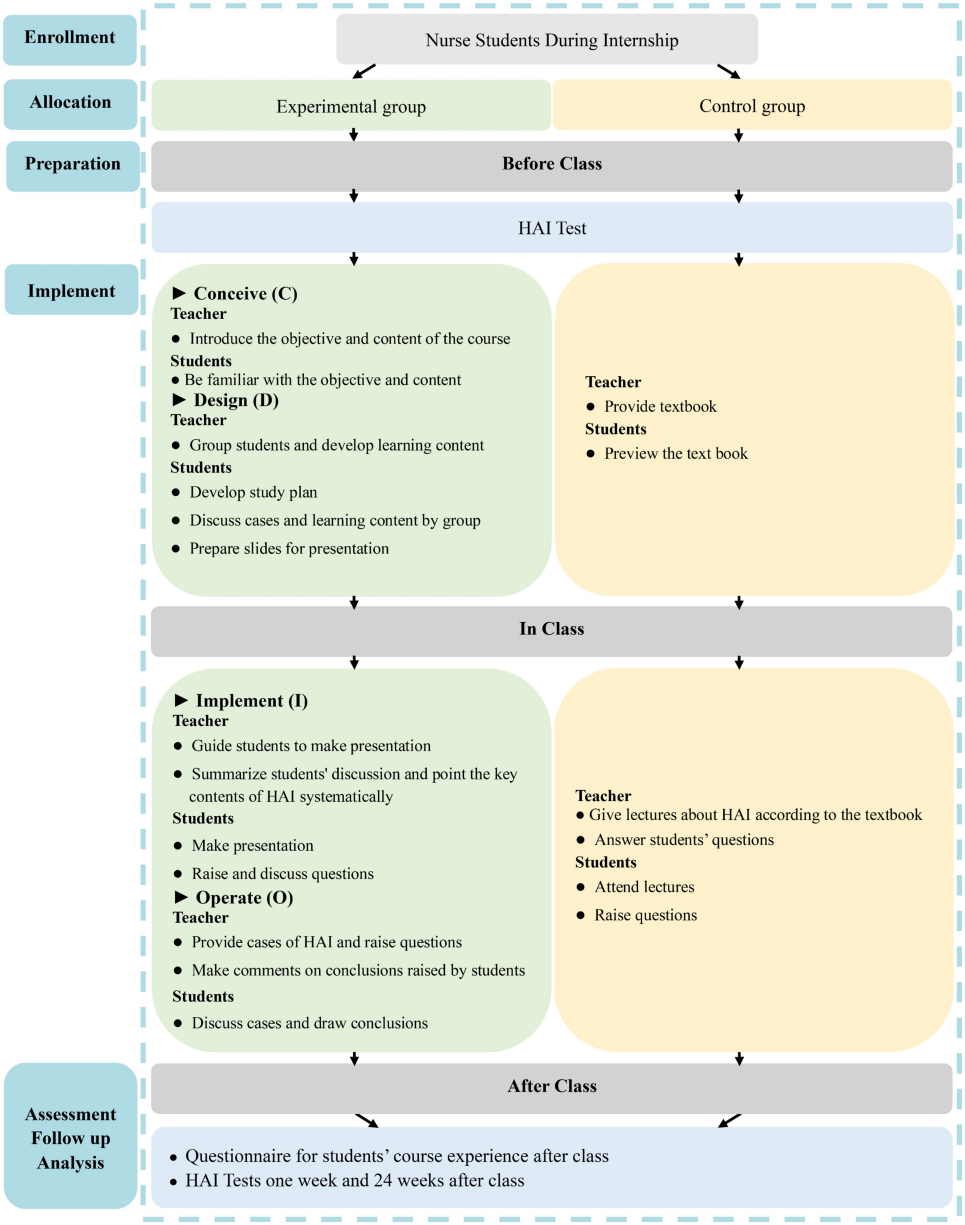
Study design. Control group: traditional LBL method; Experimental group: CDIO method.

This course comprises 12 class sessions, with each session lasting 45 min. The detailed steps of the CDIO approach for teaching HAI are outlined in Figure 1.

**Conceive (C):** Prior to the session, the instructor presents typical HAI cases and poses questions such as: “What type of HAI is this?” and “What preventive measures are recommended?.” These questions introduce students to the course content and objectives, encouraging active engagement. The learning outcomes focus on understanding HAI definitions and classifications, mastering prevention methods, and learning management strategies for post-occurrence situations.

**Design (D):** Instructors allocate students into cohorts of nine to 10 members, with each group selecting a leader. The groups are tasked with discussing cases, creating learning strategies, and preparing PowerPoint presentations. The design phase aims to stimulate collaborative learning and ensure that students are prepared for in-depth discussions during class.

**Implement (I):** During class, instructors guide students in case analysis using provided course materials and additional resources. Students present their findings via PowerPoint and receive feedback from the instructor. The focus is on facilitating collaborative problem-solving and enhancing understanding through group discussions.

**Operate (O):** In this phase, real hospital cases are used to help students review key concepts. Students are encouraged to share their perspectives, which are then evaluated and refined through real-time feedback from the instructor. This process ensures that students fully grasp the key concepts of HAI management and prevention.

#### The LBL approach for HAI curriculum

The LBL approach for the HAI curriculum consisted of 12 sessions, with each session enduring 45 min. Before the session, the instructor supplied an HAI textbook to the students for preliminary review. During the course, a 40-min lecture was delivered to clarify aspects of HAI, succeeded by a brief Q&A segment lasting about 5 min (Figure 1).

### Outcomes measures

#### Evaluation of teaching effect

##### Test design framework

To evaluate students’ comprehension and application of knowledge, we designed repeated measured tests including a pre-course test and two post-course assessments, conducted immediately post-training, 1 week later, and at 24 weeks.

**Question Categories and Scoring**

In line with Bloom’s Taxonomy, the examination questions were divided into two categories: fundamental theoretical knowledge (25 points) and case analysis (25 points). The theoretical knowledge segment consisted of 25 multiple-choice questions, each worth one point. The case analysis section comprised 25 HAI case questions, also valued at one point each. Each test had a total score of 50 points and was allotted a duration of 60 min.

##### Test objectivity and feedback

The test questions administered to the students at different times were different; however, the difficulty level of the questions across the three tests was consistent. The difficulty level was verified for consistency by two different educators. All the questions in the tests were multiple-choice, with a unique correct answer for each question, making them objective questions. Within 1 week after each test, each student participating in the study privately received the correct answers to the questions and their scores.

### Evaluation of students’ course experience

#### Evaluation tool

To evaluate students’ perceptions of the course, a questionnaire was administered upon completion of the curriculum. The study utilized the CEQ, a widely recognized and dependable tool used to assess students’ perceptions of teaching quality and to explore their course experiences (17).

#### Reliability and validity

A substantial body of evidence in the literature supports the reliability and validity of the CEQ, indicating that it has a relatively high level of reliability and validity in assessing students’ experiences with several teaching methods (17, 18).

### Questionnaire structure

This questionnaire consists of 36 questions that cover six key characteristics of the learning environment (Table 1). These dimensions provide a comprehensive evaluation of the course from the students’ perspective.

**Table 1:** Constructs and items of course evaluation questionnaire (CEQ).

Constructs	Related items in CEQ
Good Teaching (GT) scale	Q4. The teaching staff of this course motivate students to do their best work Q9. Staff here put a lot of time into commenting on students’ work Q20.The staff make a real effort to understand difficulties students may be having with their work Q22.Teaching staff here normally give helpful feedback on how you are going Q23.Our lecturers are extremely good at explaining things to us Q25.Teaching staff here work hard to make subjects interesting Q31.Staff show no real interest in what students have to say Q33.This course really tries to get the best out of all its students
Clear Goals and Standards (CG) scale	Q1. It’s always easy here to know the standard of work expected Q8. You usually have a clear idea of where you are going and what’s expected of you Q18.It’s often hard to discover what’s expected of you in this course Q24.The aims and objectives of this course are NOT made very clear Q35.The staff here make it clear right from the start what they expect from students
Appropriate Assessment (AA) scale	Q7. Lecturers here frequently give the impression they have nothing to learn from students Q10.To do well on this course all you really need is a good memory Q17.Staff seem more interested in testing what you have memorized than what you have understood Q26.Too many staff ask us questions just about facts Q29.Feedback on student work is usually provided ONLY in the form of marks and grades Q32.It would be possible to get through this course just by working hard around exam times
Appropriate Workload (AW) scale	Q5. The workload is too heavy Q14.It seems to me that the syllabus tries to cover too many topics Q19.We are generally given enough time to understand the things we have to learn Q27.There’s a lot of pressure on you as a student here Q36.The sheer volume of work to be got through in this course means you cannot comprehend it all
Generic Skills (GS) scale	Q2. This course has helped me to develop my problem-solving skills Q6. This course has sharpened my analytic skills Q11.This course has helped develop my ability to work as a team member Q12.As a result of doing this course, I feel more confident about tackling unfamiliar problems Q13.This course has improved my written communication skills Q28.This course has helped me develop the ability to plan my own work
Emphasis on Independence (IN) scale	Q3. There are few opportunities to choose the particular areas you want to study Q15.The course has encouraged me to develop my own academic interests as far as possible Q16.Students have a great deal of choice over how they are going to learn in this course Q21.Students here are given a lot of choice in the work they have to do Q30.We often discuss with our lecturers or tutors how we are going to learn in this course Q34.There’s very little choice in this course in the ways you are assessed

#### Response Scale

Responses of this questionnaire were gaged using a 5-point Likert scale, where 5 represented very satisfied/strongly agree, 4 indicated satisfied/agree, 3 denoted neutral, 2 suggested dissatisfied/disagree, and 1 signified very dissatisfied/strongly disagree (17).

### Randomization and blinding

Participants were randomly assigned to one of two intervention groups: the traditional LBL method or the CDIO model. A computer-generated random number sequence was used to ensure random allocation. The randomization process was performed by an independent statistician who was not involved in any other aspect of the study. Due to the involvement of course instruction, blinding was not possible for participants and educators. However, blinding was applied to data analysts, exam graders, and those responsible for analyzing the questionnaire results.

### Statistical analysis

Normality of the data was assessed using the Shapiro–Wilk test. Data were expressed as either mean ± standard deviations (SDs) or median values with interquartile ranges (IQR), depending on the distribution characteristics. Age comparisons were conducted using the Mann–Whitney U test. Pre-training HAI test scores of both groups were evaluated using the t-test. For assessing HAI test scores at different intervals, repeated measures analysis of variance (ANOVA) was utilized. The Mann–Whitney U test was employed to analyze the 5-level Likert scale ratings regarding students’ assessment of course experience. Statistical analyses were performed using SPSS version 26.0 (SPSS Inc., Chicago, United States). All statistical tests were two-tailed, with a significance threshold set at *p* < 0.05.

## Results

### Baseline data of study subjects

This study involved 110 female third-year nursing students participating in internships with no previous knowledge about HAI who were randomly allocated into two groups. The experimental group, consisting of 55 students, adopted the CDIO approach, while the control group, initially comprising 55 students, employed the LBL method. During the study, the control group experienced a loss of one participant, resulting in 54 remaining members. Prior to initiating the curriculum, comparative analysis of variables such as age and baseline HAI test scores revealed no statistically significant differences between the groups (*p* > 0.05; Table 2).

**Table 2:** Baseline of enrolled nursing students.

Characteristics	Control group (*n* = 54)	Experimental group (*n* = 55)	z/t	*p*
Age (Yrs)	20.0 (20.0–21.0)	20.0 (20.0–21.0)	−0.302	0.762^a^
Total test scores before training	24.52 ± 3.02	24.93 ± 3.03	−0.641	0.523^b^
Theoretical test scores before training	15.56 ± 2.60	15.51 ± 2.72	0.910	0.928^b^
Application test scores before training	9.00 ± 1.90	9.42 ± 2.03	−1.109	0.270^b^

a The two groups were compared using a Mann–Whitney U test.

b The two groups were compared using an independent sample *t*-test.

#### HAI test results.

The results of the HAI test show significant improvements in the experimental group compared to the control group following the intervention. Baseline scores were similar for both groups. After 1 week of intervention, the experimental group exhibited substantial gains in total scores, theoretical knowledge, and practical application, as well as the control group. Notably, the experimental group outperformed the control group in both overall performance and practical application scores. At 24 weeks post-training, the experimental group maintained higher scores across all areas, with significant differences in overall performance, theoretical knowledge, and practical application compared to the control group.

**Figure 2:**
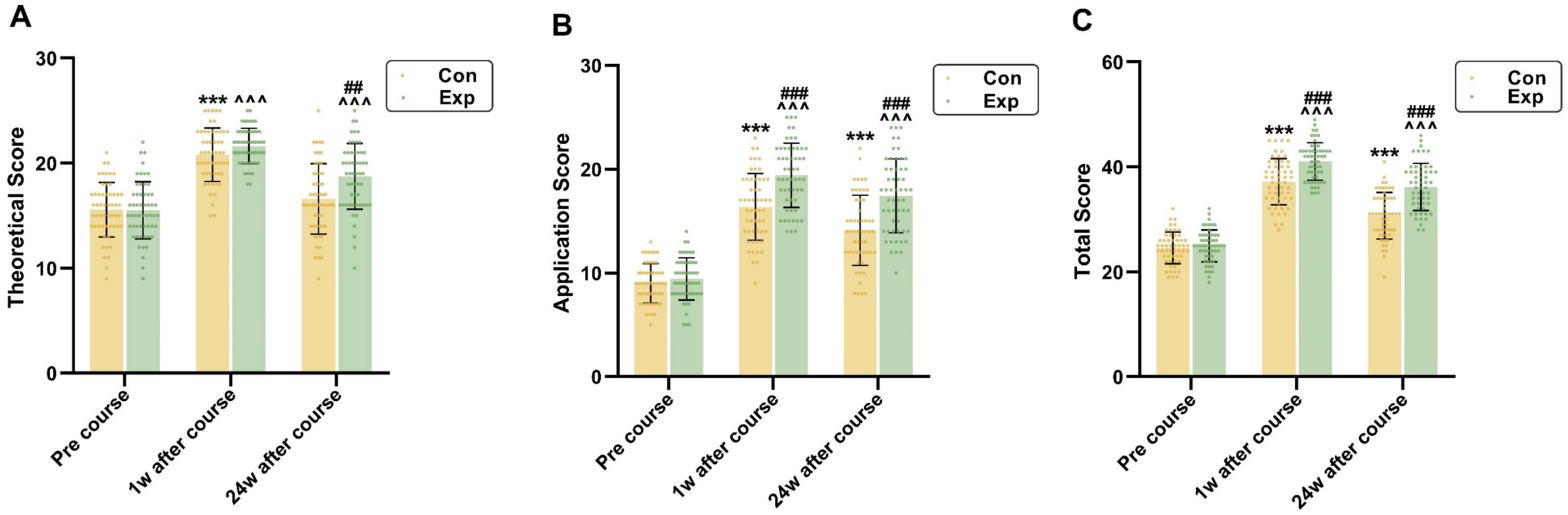
HAI tests scores in Con (*n* = 54) and Exp (*n* = 55) groups at different time points. **(A)** Theoretical Score; **(B)** Application Score; **(C)** Total Score. Con: control group with the traditional LBL method. Exp: experimental group with the CDIO method. Data are presented as mean ± standard deviation (SD). ***p < 0.001 vs. pre-course in Con group, ^^^*p* < 0.001 vs. pre-course in Exp group, ###*p* < 0.001 vs. application and total scores in Con group at 1w and 24w after course, ## *p* < 0.01 vs. theoretical scores in Con group at 24w after course.

Figure 2 displays the HAI test results for both study groups. One week before starting the HAI curriculum, a preliminary assessment was conducted. In the experimental group, the average total score recorded was 24.92 ± 3.03, with scores for theoretical knowledge at 15.51 ± 2.72 and for practical application at 9.42 ± 2.03. Meanwhile, the control group exhibited mean scores of 24.56 ± 3.02 overall, 15.56 ± 2.60 in theoretical knowledge, and 9.00 ± 1.90 in application. Statistical analysis showed no significant differences between the two groups in these scores (*p* > 0.05), suggesting equivalent baseline characteristics.

One week post-intervention, significant enhancements were observed in the scores of the experimental group across all metrics: total scores escalated from 24.92 ± 3.03 to 41.02 ± 3.57, theoretical knowledge from 15.51 ± 2.72 to 21.62 ± 1.69, and practical application from 9.42 ± 2.03 to 19.40 ± 3.09 (*p* < 0.001 for all). The control group, employing traditional LBL methods, also showed increases in these areas: total scores rose from 24.56 ± 3.02 to 37.15 ± 4.40, theoretical knowledge from 15.56 ± 2.60 to 20.80 ± 2.55, and application scores from 9.00 ± 1.90 to 16.35 ± 3.22 (*p* < 0.001 for all). Post-training comparisons indicated that the experimental group outperformed the control group in total and application scores (41.02 ± 3.57 vs. 37.15 ± 4.40 and 19.40 ± 3.09 vs. 16.35 ± 3.22, respectively, *p* < 0.001). While the experimental group also showed a higher mean score in theoretical knowledge (21.62 ± 1.69 vs. 20.80 ± 2.55), the difference was marginally non-significant (*p* = 0.050).

At 24 weeks following the completion of the training program, participants in the experimental group achieved mean scores of 36.16 ± 4.51 for overall performance, 18.75 ± 3.14 for theoretical knowledge, and 17.42 ± 3.55 for practical application. In contrast, the control group registered mean scores of 30.69 ± 4.43 overall, 16.59 ± 3.36 for theoretical knowledge, and 14.09 ± 3.38 for practical application. Notable differences were evident between the groups regarding both overall and practical application scores at the 24-week mark, showing high statistical significance (*p* < 0.001). Additionally, scores for theoretical knowledge exhibited statistically significant differences (*p* < 0.01).

### Comparison of students’ course experience

**Figure 3:**
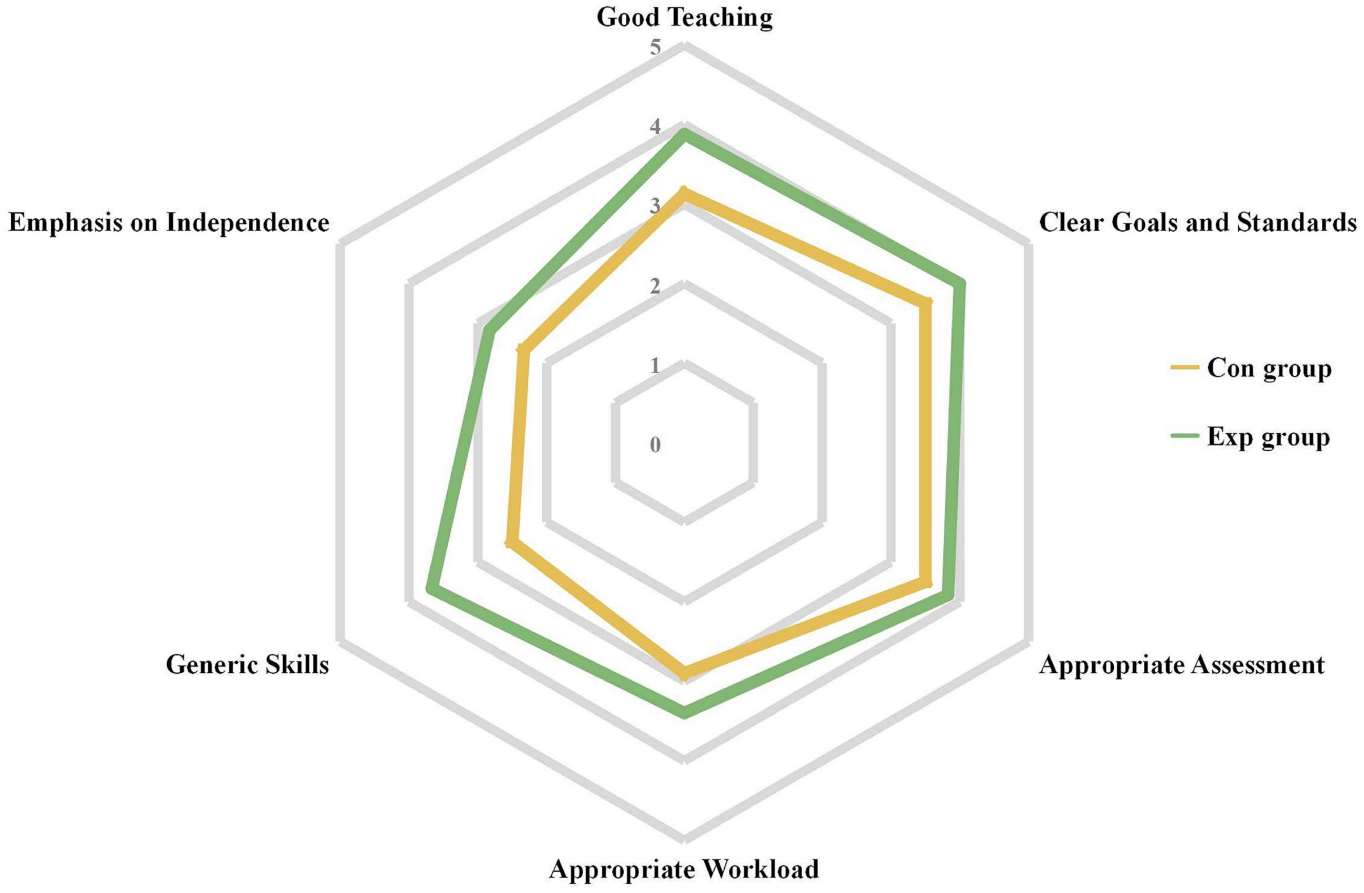
Overall of students’ course experience in Con (*n* = 54) and Exp (*n* = 55) groups. Con, control group with the traditional LBL method. Exp, experimental group with the CDIO method.

In this study, all 109 distributed questionnaires were returned, resulting in a 100% response rate. Survey results indicated that the experimental group had higher satisfaction with teaching quality, goal clarity, assessment appropriateness, skill development, and independence promotion (Figure 3). Specifically, the experimental group reported improvements in teaching (*p* < 0.001), goal clarity (*p* = 0.002), assessment appropriateness (*p* = 0.008), generic skills (*p* < 0.001), and emphasis on independence (*p* < 0.001). However, students in the experimental group reported an increase in workload, as evidenced by statistical significance (*p* < 0.001; Table 3).

**Table 3:** Comparison of students’ course experience between Con and Exp groups.

Constructs	Items in CEQ	Con group	Exp group	*z*	*p*
Good Teaching (GT) scale	Overall	3.13 (2.75–3.53)	3.88 (3.50–4.13)	−5.648	<0.001
	Q 4	3.00 (3.00–4.25)	4.00 (3.00–5.00)	−1.979	0.048
	Q 9	3.00 (3.00–4.00)	4.00 (4.00–5.00)	−4.396	< 0.001
	Q20	3.00 (3.00–4.00)	4.00 (3.00–5.00)	−2.324	0.020
	Q22	3.00 (2.00–4.00)	4.00 (3.00–5.00)	−3.087	0.002
	Q23	3.00 (2.00–4.00)	4.00 (3.00–4.00)	−1.936	0.053
	Q25	2.50 (2.00–3.00)	4.00 (3.00–5.00)	−6.219	< 0.001
	Q31	3.00 (2.00–4.00)	4.00 (3.00–5.00)	−4.313	< 0.001
	Q33	3.00 (2.00–4.00)	4.00 (3.00–4.00)	−4.015	< 0.001
Clear Goals and Standards (CG) scale	Overall	3.50 (2.95–4.05)	4.00 (3.60–4.40)	−3.138	0.002
	Q 1	3.00 (3.00–4.00)	4.00 (3.00–4.00)	−2.187	0.029
	Q 8	3.00 (2.00–4.00)	4.00 (3.00–5.00)	−2.640	0.008
	Q18	3.50 (3.00–4.00)	4.00 (4.00–5.00)	−3.112	0.002
	Q24	4.00 (3.00–4.00)	4.00 (3.00–5.00)	−2.078	0.038
	Q35	4.00 (3.00–4.00)	4.00 (3.00–5.00)	−2.463	0.014
Appropriate Assessment (AA) scale	Overall	3.50 (2.83–4.04)	3.83 (3.50–4.33)	−2.657	0.008
	Q 7	3.00 (3.00–4.00)	4.00 (3.00–4.00)	−2.479	0.013
	Q10	3.00 (2.75–4.00)	4.00 (3.00–5.00)	−3.133	0.002
	Q17	4.00 (3.00–5.00)	4.00 (3.00–5.00)	−0.849	0.396
	Q26	3.00 (2.75–4.00)	4.00 (3.00–4.00)	−3.456	0.001
	Q29	4.00 (3.00–4.00)	4.00 (3.00–4.00)	−0.897	0.370
	Q32	4.00 (3.00–5.00)	4.00 (3.00–5.00)	−0.677	0.498
Appropriate Workload (AW) scale	Overall	2.90 (2.40–3.40)	3.40 (2.80–4.20)	−3.312	0.001
	Q 5	3.00 (2.00–3.00)	4.00 (3.00–4.00)	−4.527	< 0.001
	Q14	3.00 (2.00–3.00)	3.00 (2.00–4.00)	−0.894	0.371
	Q19	3.00 (2.00–4.00)	4.00 (3.00–4.00)	−2.322	0.020
	Q27	3.00 (2.00–4.00)	4.00 (3.00–5.00)	−3.859	< 0.001
	Q36	3.00 (2.00–3.00)	3.00 (2.00–4.00)	−1.461	0.144
Generic Skills (GS) scale	Overall	2.50 (2.00–2.67)	3.67 (3.00–4.00)	−7.718	< 0.001
	Q 2	2.00 (2.00–3.00)	4.00 (3.00–5.00)	−7.466	< 0.001
	Q 6	2.50 (2.00–3.00)	4.00 (3.00–5.00)	−7.185	< 0.001
	Q11	2.00 (2.00–3.00)	4.00 (3.00–5.00)	−7.565	< 0.001
	Q12	3.00 (2.00–3.00)	4.00 (3.00–4.00)	−5.169	< 0.001
	Q13	3.00 (2.00–3.00)	3.00 (2.00–3.00)	−1.116	0.265
	Q28	2.00 (2.00–3.00)	4.00 (3.00–4.00)	−6.245	< 0.001
Emphasis on Independence (IN) scale	Overall	2.33 (2.00–2.71)	2.83 (2.50–3.17)	−3.979	< 0.001
	Q 3	2.00 (1.75–2.00)	3.0 0 (2.00–3.00)	−4.727	< 0.001
	Q15	3.00 (2.00–3.00)	3.00 (3.00–4.00)	−3.906	< 0.001
	Q16	2.00 (2.00–3.00)	3.00 (2.00–3.00)	−1.349	0.177
	Q21	2.00 (2.00–3.00)	3.00 (2.00–3.00)	−1.631	0.103
	Q30	2.50 (2.00–3.00)	3.00 (3.00–4.00)	−3.874	< 0.001
	Q34	2.00 (2.00–3.00)	3.00 (2.00–3.00)	−1.689	0.091

Con, control group with the traditional LBL method; Exp, experimental group with the CDIO method; CEQ, Course Evaluation Questionnaire.

## Discussion

HAI poses a significant threat to global healthcare systems, impacting millions of patients annually, leading to substantial medical costs and increased mortality rates (19). The COVID-19 pandemic reshaped healthcare services, emphasizing the importance of preventing HAIs, which posed heightened risks to patients and healthcare workers due to increased susceptibility, making infection control practices crucial (20, 21).During this period, strict infection control measures implemented in medical environments and hospitals, including respiratory hygiene and hand hygiene, can significantly reduce the incidence density of multidrug-resistant bacteria (22). Similarly, another study observed the impact of infection prevention and control measures on HAI and related outcomes in patients with cirrhosis. The study found that the infection prevention and control program reduced the incidence of HAI by nearly 50% (23). The studies suggest that strict preventive control strategies will effectively reduce the incidence of HAI.

Nursing plays a crucial role in the prevention and control of HAI. A study indicated that patients exposed to nurses with limited work experience had an increased risk of bloodstream infections compared to patients not exposed to such nurses. Additionally, nurse understaffing, measured as low nursing hours relative to target hours, was associated with an increased risk of surgical-site infections (24). Furthermore, research shows that nurses’ knowledge and skills are crucial for preventing HAI, with effective training significantly reducing central venous catheter-related infections by enabling high-quality care (25). These studies suggest that enhancing nurses’ knowledge and practical skills regarding HAI can effectively reduce the incidence of HAI and improve healthcare quality.

LBL is the most common teaching method in nursing education, widely used for its advantages in the systematic and efficient delivery of theoretical knowledge (26).

Recent research has indicated some shortcomings in the application of LBL in nursing education, prompting us to continuously explore new teaching methods to enhance the quality of nursing education (27). The CDIO model originated in the late 1990s and was first used in the field of engineering education. The CDIO model emphasizes active student participation, encouraging them to engage in hands-on practices and problem-oriented learning; it aims to cultivate students who not only possess solid theoretical knowledge but also know how to apply this knowledge in practical contexts (28). The advantages of the CDIO model in the field of medical and nursing education compared to traditional teaching methods have been confirmed by multiple studies (14–16). Our research is similar to the studies mentioned above; the CDIO model allows students to significantly outperform the control group in terms of the overall scores and practical application scores. This advantage may be due to students actively participating in teaching activities and learning from practical cases.

After the course, the long-term retention of theoretical knowledge and practical skills is also a direction that research on new teaching methods should focus on (29).

Previous research has shown that active learning modalities have an advantage over traditional LBL teaching models in terms of long-term knowledge retention (30).

Team-Based Learning (TBL) is an active learning strategy, and research has shown that it is more effective than the traditional LBL model in enhancing the long-term retention of knowledge about postpartum hemorrhage (PPH) among Indonesian midwifery students (31). The advantages of flipped classroom combined with situational simulation in nursing education for long-term knowledge retention among students have also been observed (32). Our research shows that the CDIO teaching model significantly outperforms the LBL model in terms of long-term retention of theoretical knowledge and practical application. The teaching methods that stand out in these areas share some common characteristics: (1) They are student-centered, encouraging active participation; (2) They promote interaction with peers and teachers to enhance understanding; (3) They emphasize practice and hands-on activities over traditional LBL, helping students apply theory in real-world scenarios.

Extensive evidence in the literature confirms the reliability and validity of CEQ in the context of higher education (17, 18, 33, 34). Recently, this questionnaire has also been applied in medical and nursing education (35, 36). We found that the CDIO model significantly enhances students’ identification of the “Clear Goals and Standards” and “Good Teaching” aspects of the course, which is consistent with previous research in nursing education (37). This is because the CDIO model first provides students with clear learning objectives during the “Conceive” stage. This upfront design ensures that students have a clear understanding of the goals they need to achieve throughout the course.

In teaching activities, teams need to collaborate in analyzing problems and exploring solutions. This process encourages students to utilize the collective intelligence of the team, thereby enhancing team collaboration, which helps them perform at their best. Simultaneously, students work in groups for discussions and presentations, with teacher guidance and feedback strengthening the overall interest of the course. All of these elements form the foundation of “Good Teaching.” “Good Teaching” has also been observed in other new student-centered teaching models (38, 39).

The CDIO model enables students to demonstrate higher levels of “generic skills” in their course experience, including teamwork, communication, innovation, critical thinking, and the ability to solve problems independently. The CDIO teaching model emphasizes practice-oriented learning, developing students’ knowledge application and skills through problem-solving in real-world cases (40). This approach likely makes students more perceptive and accurate in assessments that value both theory and practice, as they engage more in hands-on activities and reflect on their learning process.

“Emphasis on Independence” is an advantage of the CDIO model: it emphasizes the integration of knowledge and skills by students in real-world contexts. In the “Operate” phase, the application of case studies is a highly practical learning process that encourages students to actively learn, explore new knowledge, and make independent decisions when solving real problems. Our study found that the CDIO model increases students’ workload because in the CDIO model, students are more involved in teaching activities, such as group discussions and preparing slides, whereas in LBL, students only passively receive knowledge. This increase in learning load has also been found in other student-centered new teaching methods (41). Therefore, when applying the CDIO model, educators need to carefully consider how to enhance educational outcomes while reasonably allocating students’ learning tasks, in order to avoid excessively increasing their workload.

### Limitation

This study presents several limitations that impact the generalization of its findings. Firstly, the use of small, single-center sample sizes limits the robustness and wider applicability of the results. Secondly, the study is limited to a mid-term follow-up period, making it difficult to assess the longer-term retention effects and effectiveness of the educational interventions. Future research should consider using larger, multi-center samples, longer and more comprehensive training periods, and diversified group assignments to enhance the reliability and applicability of the results.

## Conclusion

Our study has shown that applying the CDIO framework to nursing education in HAI courses enhances students’ practical application skills and promotes sustained retention in this area. Although further studies are needed to minimize potential biases and evaluate its applicability to a broader audience, preliminary results indicate that the CDIO model leads to significant improvements in good teaching, clear goals and standards, appropriate assessment, generic skills, and emphasis on independence compared to the control group, though they also reported a statistically significant increase in workload.

## Data Availability

The original contributions presented in the study are included in the article/supplementary material, further inquiries can be directed to the corresponding authors.
